# Influence of fluid balance on the prognosis of patients with sepsis

**DOI:** 10.1186/s12871-021-01489-1

**Published:** 2021-11-05

**Authors:** Luming Zhang, Fengshuo Xu, Shaojin Li, Xiaoyu Zheng, Shuai Zheng, Hui Liu, Jun Lyu, Haiyan Yin

**Affiliations:** 1 Intensive Care Unit, The First Affiliated Hospital of Jinan University, 510630 Guangzhou, Guangdong Province People’s Republic of China; 2 Department of Clinical Research, The First Affiliated Hospital of Jinan University, Guangzhou, Guangdong Province China; 3 School of Public Health, Xi’an Jiaotong University Health Science Center, Xi’an, Shaanxi Province China; 4 Department of Orthopaedics, The First Affiliated Hospital of Jinan University, Guangzhou, Guangdong Province China; 5 School of Public Health, Shannxi University of Chinese Medicine, Xianyang, Shaanxi Province China

**Keywords:** sepsis, eICU-CRD, Fluid balance, Prognosis

## Abstract

**Background:**

Early and timely fluid treatment or resuscitation are the basic measures for the active treatment of sepsis. Our aim is to further explore the relationship between fluid balance and prognosis in patients with sepsis on a daily basis for 5 days.

**Methods:**

Sepsis patients in eICU Collaborative Research Database were divided into the negative balance group (NB/−) and the positive balance group (PB/+) according to daily fluid balance. The primary outcome was in-hospital mortality. Survival differences between the groups were analyzed by using Cox regression. Then dose-response relationship between fluid balance and in-hospital mortality was studied using restricted cubic splines (RCSs). Furthermore, patients with fluid balance data for the previous three consecutive days were selected and divided into eight groups (“+/+/+”, “+/+/−”, “+/ −/−”, “+/ −/+”, “−/ −/−”, “−/ −/+”, “−/+/+”, and “−/+/−”). Kaplan–Meier curves and Cox regression were used to show the survival difference between groups.

**Results:**

Our study, which included 19,557 patients in a multicenter database, showed that positive fluid balances on days 1, 2, and 3 after sepsis diagnosis were associated with poor prognosis with the HRs of 1.29 (1.20,1.40), 1.13 (1.01,1.27), and 1.25 (1.08,1.44), respectively, while the fluid balance on days 4 and 5 had no effect on the primary outcome. Then RCSs showed an overall trend that the risk of in-hospital mortality on days 1, 2, and 3 increased with increasing fluid balance. For three consecutive days of fluid balance, we studied 9205 patients and Kaplan–Meier curves revealed survival differences among patients in the eight groups. The cox model demonstrated that compared with the “+/+/+” group, the “+/ −/−”, “−/ −/−”, “−/ −/+”, “−/+/+”, and “−/+/−” groups had a lower risk of in-hospital mortality, with HRs of 0.65 (0.45,0.93), 0.72 (0.60,0.86), 0.63 (0.43,0.93), 0.69 (0.48,0.98), and 0.63 (0.42,0.96), respectively.

**Conclusions:**

In patients with sepsis, positive fluid balance on days 1, 2, and 3 was associated with adverse outcomes. For patients with fluid balance for three consecutive days, the “+/−/−”, “−/ −/−”, “−/−/+”, “−/+/+”, and “−/+/−” groups were less likely to die in hospital than the “+/+/+” group.

## Background

Sepsis is defined as life-threatening organ dysfunction caused by a host’s dysfunctional response to infection and is associated with a high incidence of morbidity and mortality worldwide [1]. Despite the use of multiple antibiotics and organ support therapy, the mortality rate of this dysfunction remains high. The release of bacterial toxins, inflammatory mediators, cytokines, and vasoactive substances caused by infection can increase capillary permeability and lead to extensive plasma extravasation, thus resulting in insufficient effective circulating blood volume, microcirculation dysfunction, electrolyte disturbance and acidosis, and other internal environmental changes [2]. Therefore, sepsis is an important cause of death in emergency departments and intensive care units (ICUs) [3].

Early and timely fluid treatment or resuscitation and necessary vasopressor use are the basic measures for the active treatment of sepsis. Among these methods, fluid therapy or resuscitation aims to correct the relative or absolute deficiency of blood volume through rapid fluid supplementation, to ensure normal cardiac output and organ blood perfusion, and to protect organ function [4]. However, the correct guidance of fluid therapy remains a difficult issue due to the clinical signs of fluid response and the limitations of monitoring techniques.

In recent years, a growing body of evidence has shown that over-aggressive fluid resuscitation may have side effects. For example, a previous work [5] demonstrated that the persistence of positive daily fluid balance over time is strongly associated with high mortality in patients with sepsis. However, the Save Sepsis Campaign states that in the treatment of patients with severe sepsis or septic shock, intravenous access should first be established and aggressive fluid resuscitation should be initiated and that strict adherence to three and six bunching regimens can improve outcomes [6]. Moreover, with the further revision of the “1 h bundle for sepsis” in 2018, volume overload is increasing in patients with sepsis [7].

The possibility of volume overload due to the improper infusion of large amounts of fluids is increasingly recognized as an independent risk factor for disability and death in critical illness [8]. A prospective, multicenter, observational study revealed that high fluid volume is associated with reduced mortality in patients with shock lasting for 3 days or longer [9]. We used a large multicentric eICU Collaborative Research Database to explore the relationship between fluid balance and prognosis in patients with sepsis within 5 days at different time points to further investigate this issue.

### Methods

## Data source

The data analyzed in this study were collected from the eICU Collaborative Research Database (eICU-CRD), a public, multicenter ICU database that includes electronic medical records from 208 hospitals and data from more than 200,000 patients in 2014−2015 [10, 11]. All information related to the patient’s identity is hidden. Therefore, informed consent does not need to be obtained from the patient. The data research training of the cooperative organization training program was completed, and database permissions were obtained. All the data were collected from the physical network’s official website (https://eicu-crd.mit.edu/).

### Study population

Sepsis was diagnosed by using the latest criteria for sepsis 3 [12], which is defined as a life-threatening infection combined with an acute increase in Sequential Organ Failure Assessment score (SOFA ≥ 2).

Therefore, we extracted the information of infected patients with SOFA ≥ 2 from the eICU-CRD, among which 36,302 patients met the diagnostic criteria for sepsis 3. Exclusion criteria were as follows: patients < 18 years of age, patients who died within 24 h of admission to the ICU, and patients without fluid records. A total of 19,557 patients were included in this study.

### Data extraction

We use SQL (Structured Query Language) for data extraction. The patientunitstayid identifier of the patients with sepsis was used to extract the general information of the patient, including age, gender, weight, height, and ICU type; intervention measures: dialysis, ventilator, and vasopressor; comorbidity: stroke, congestive heart failure (CHF), hypertension, chronic obstructive pulmonary disease (COPD), renal failure, liver diseases, diabetes, and cancer; severity scores, namely, acute physiology and chronic health evaluation scoring system (APACHE) IV and Sequential Organ Failure Assessment (SOFA) scores; infection source; and the number of patients in and out 5 days after the diagnosis of sepsis. Day 1 was defined as 24 h after the diagnosis of sepsis. The daily fluid intake is calculated as the sum of all intravenous and oral fluids. The daily output is calculated as the sum of urine output, stool volume, emesis, blood loss, dialysis ultrafiltrate yield, drainage fluid volume, puncture fluid volume (e.g. ascites, pleural fluid) etc. The invisible losses of liquids were not taken into account because they were difficult to estimate. The daily fluid balance was determined as the difference between the total intake and the total output and was divided into the negative balance group (NB/−) and the positive balance group (PB/+) on the basis of the difference.

The primary outcome was in-hospital mortality, and the secondary outcome was duration of ventilator use.

### Statistical analysis

Categorical variables were described as frequency and percentage values, and differences between the two groups were determined by using the chi-square or Fisher exact test. The Shapiro–Wilk test was used to test whether continuous variables fit the normal distribution. Continuous variables that fit the normal distribution were described as mean and standard deviation values, whereas those that did not fit the normal distribution were described as median and quaternary range values.

Cox regression was used to compare daily survival differences between the two groups. The hazard ratio (HR) and 95 % confidence interval (CI) were calculated by using multivariate Cox regression by controlling for the following confounders: age, gender, weight, height, unit type, dialysis, ventilator, vasopressor; comorbidities: stroke, CHF, hypertension, COPD, renal failure, liver disease, diabetes, and cancer; APACHE IV and SOFA scores; and infection source.

After preliminary analysis, the fluid balances on days 1, 2, and 3 were found to have an influence on the in-hospital mortality. Therefore, we conducted further analysis. The RCSs was used to explore the dose-response relationship between fluid balance on and in-hospital mortality in sepsis patients on days 1, 2, and 3. Furthermore, patients with fluid balance data for the previous three consecutive days were selected and grouped. Kaplan–Meier curves were used for survival analysis, and Cox proportional hazard regression models were used to examine the effects of various factors on hospital mortality.

All statistical analyses were conducted on R (version 4.0.3). A two-sided p-value of <0.05 was considered statistically significant.

## Results

### Baseline characteristics

**Fig. 1:**
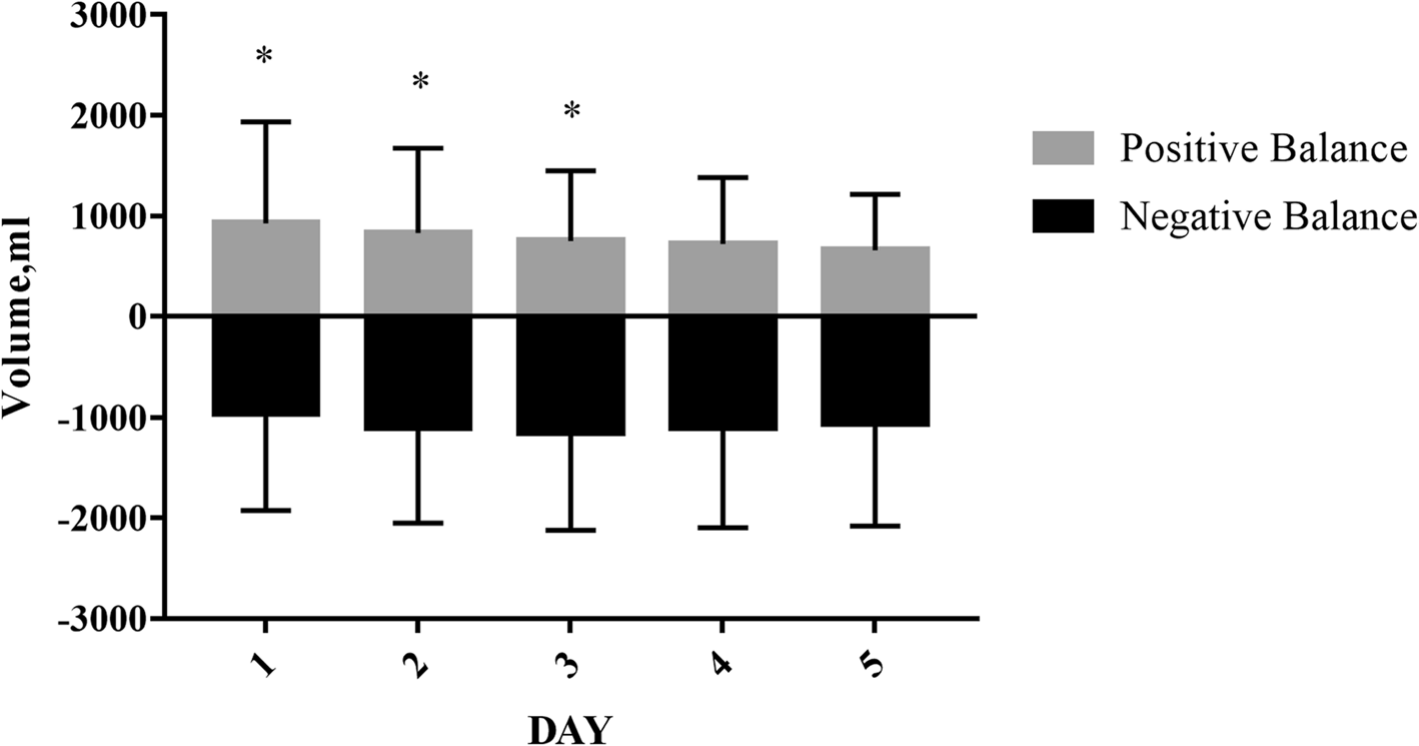
The median fluid balance volumes on days 1 to 5

A total of 19,557 patients were included in this study. Table 1 describes the baseline characteristics of the patients within first day of sepsis diagnosis. The age of patients in the NB group was lower than that in the PB group (66.00 [54.00, 77.00] vs. 68.00 [56.00, 79.00]). Males accounted for 52.8 % and 51.7 % of the patients in the NB and PB groups, respectively. The APACHE IV and SOFA scores of the NB group were lower than those of the PB group (64.00 [49.00, 81.00] vs. 67.00 [52.00, 85.00] and 6.00 [4.00, 8.00] vs. 7.00 [5.00, 9.00]). The main source of infection of the two groups of patients was pulmonary infection, which accounted for 48.3 % and 42.0 % of the cases. The general characteristics of the remaining patients can be seen in Table 1. The number of patients on days 2 to 5 were 12,960, 9850, 7931, and 6286. As shown in Fig. 1, the median fluid balance volumes on days 1 to 5 of the NB group were −960.00 (−1925.00, −345.00), −1100.00 (−2050.00, −450.00), −1150.00 (−2119.25, −471.00), −1099.00 (−2095.00, −411.00), and −1060.00 (−2080.62, −425.00) ml and those of the PB group were 924.00 (366.00, 1935.00), 830.00 (358.00, 1672.00), 749.50 (335.75, 1448.75), 719.00 (339.78, 1380.00), and 660.00 (299.00, 1215.75) ml.

**Table 1:** Baseline characteristics of the study population

	Negative Balance	Positive Balance	p
	12,252	7305	
Age (year)	66.00 (54.00, 77.00)	68.00 (56.00, 79.00)	<0.001
Gender (%)			
male	6475 (52.8)	3774 (51.7)	0.112
female	5777 (47.2)	3531 (48.3)	
Height (cm)	170.00 (162.00, 177.80)	167.60 (160.00, 177.80)	<0.001
Weight (kg)	79.30 (65.40, 98.10)	77.20 (63.52, 95.50)	<0.001
Severe Score			
Apache IV	64.00 (49.00, 81.00)	67.00 (52.00, 85.00)	<0.001
Sofa	6.00 (4.00, 8.00)	7.00 (5.00, 9.00)	<0.001
Unit type (%)			
Med-Surg ICU/SICU/MICU	9934 (81.1)	5764 (78.9)	<0.001
Cardiac ICU/CCU-CTICU/CSICU/CTICU	1968 (16.1)	1222 (16.7)	
Neuro ICU	350 ( 2.9)	319 ( 4.4)	
Source of sepsis (%)			
Lung	5919 (48.3)	3065 (42.0)	<0.001
Urinary tract	2181 (17.8)	1562 (21.4)	
Abdomen	1333 (10.9)	925 (12.7)	
Skin/Bone/Joint	964 ( 7.9)	550 ( 7.5)	
Others	1855 (15.1)	1203 (16.5)	
Ventilator (%)			
no	6170 (50.4)	4177 (57.2)	<0.001
yes	6082 (49.6)	3128 (42.8)	
Vasopressor (%)			
no	9547 (77.9)	5544 (75.9)	0.001
yes	2705 (22.1)	1761 (24.1)	
Dialysis (%)			
no	11,963 (97.6)	7234 (99.0)	<0.001
yes	289 ( 2.4)	71 ( 1.0)	
Comorbidity			
Stroke (%)			
no	11,001 (89.8)	6490 (88.8)	0.040
yes	1251 (10.2)	815 (11.2)	
Congestive heart failure (%)			
no	9854 (80.4)	5963 (81.6)	0.041
yes	2398 (19.6)	1342 (18.4)	
Hypertension (%)			
no	5350 (43.7)	3479 (47.6)	<0.001
yes	6902 (56.3)	3826 (52.4)	
COPD (%)			
no	9458 (77.2)	5835 (79.9)	<0.001
yes	2794 (22.8)	1470 (20.1)	
Renal failure (%)			
no	11,449 (93.4)	6599 (90.3)	<0.001
yes	803 ( 6.6)	706 ( 9.7)	
Liver disease (%)			
no	11,869 (96.9)	6991 (95.7)	<0.001
yes	383 ( 3.1)	314 ( 4.3)	
Diabetes (%)			
no	8211 (67.0)	4865 (66.6)	0.557
yes	4041 (33.0)	2440 (33.4)	
Cancer (%)			
no	10,166 (83.0)	6021 (82.4)	0.333
yes	2086 (17.0)	1284 (17.6)	
Length of stay			
Hospital los (day)	8.29 (5.11, 14.01)	7.87 (4.82, 13.41)	<0.001
In-hospital mortality (%)			
no	10,801 (88.2)	6106 (83.6)	<0.001
yes	1451 (11.8)	1199 (16.4)	

### Cox proportional hazard regression model

**Fig. 2:**
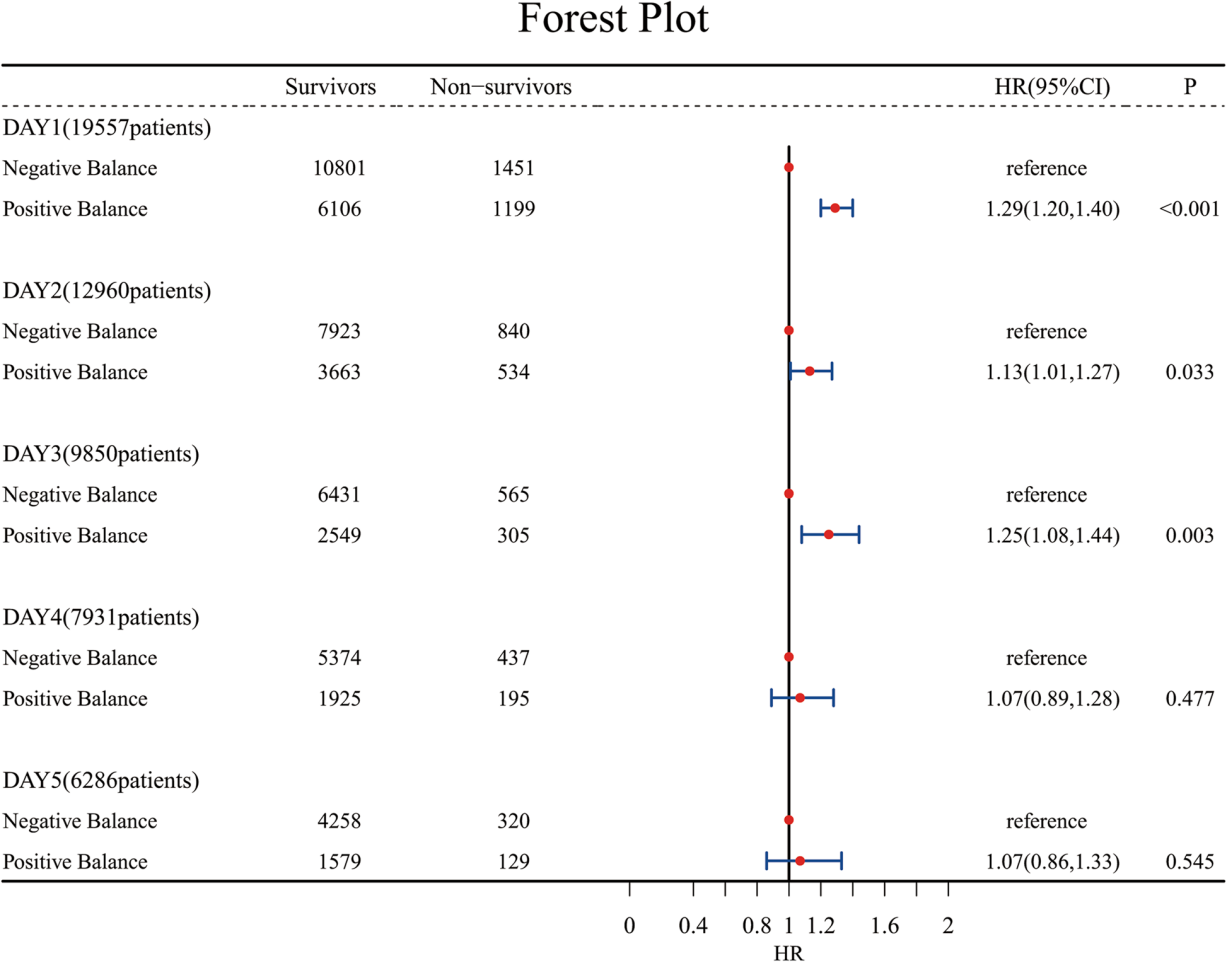
Relationship between fluid balance on days 1 to 5 and in-hospital mortality. The hazard ratios (HRs) and 95 % confidence intervals (error bars) were calculated from the COX regression, and the adjustment factors are age, gender, weight, height, unit type, dialysis, ventilator, vasopressor; comorbidities: stroke, CHF, hypertension, COPD, renal failure, liver disease, diabetes, and cancer; APACHE IV and SOFA scores; infection source

After controlling for potential confounders in Cox regression, the risk of in-hospital mortality was found to be statistically higher in the PB group than in the NB group on day 1 after the diagnosis of sepsis. The HRs (95CI%) of in-hospital mortality for the PB group were 1.29 (1.20, 1.40). This result indicated that the risk for in-hospital mortality in the PB group was 1.29 higher than that in the NB group. The same trend was observed for the risks of in-hospital mortality on days 2 and 3 after sepsis diagnosis, which were 1.13 and 1.25 times higher in the PB group than in the NB group. No significant difference in the in-hospital mortalities between the two groups on days 4 and 5 were observed (Fig. 2).

### Further analysis

**Fig. 3:**
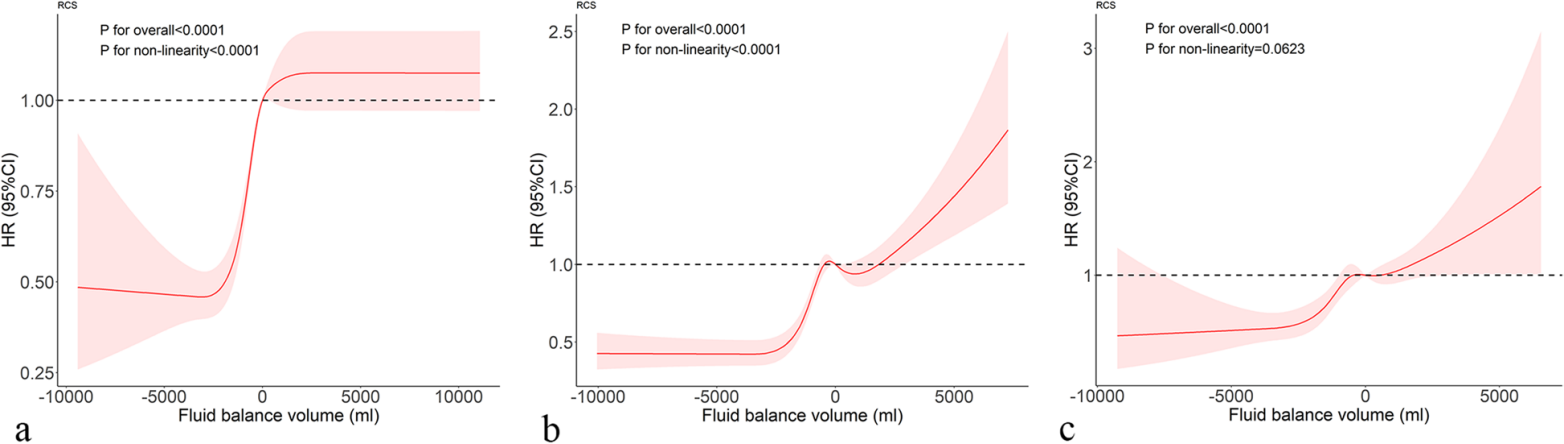
The dose-response relationship between fluid balance on day 1~3 and in-hospital mortality in sepsis patients. a, b, and c represent the first, second, and third days respectively, the adjustment factors are consistent with Fig. 2

The above results indicated that fluid balance on days 1–3 had an influence on the in-hospital mortality of patients after sepsis diagnosis. RCSs results showed a non-linear relationship between fluid balance volume and the risk of in-hospital mortality on days 1 and 2, while no such relationship was observed on day 3 (Fig. 3). On the first day, there was an “inverse Z” type relationship, between -2500ml and 1500mL, with a positive correlation between fluid volume and the risk of hospital death. There was a “W” relationship on the second day. Overall, after -2500 ml, fluid volume was positively associated with the risk of hospital death. On day 3, the overall risk of in-hospital mortality increased as fluid volume increased.

**Fig. 4:**
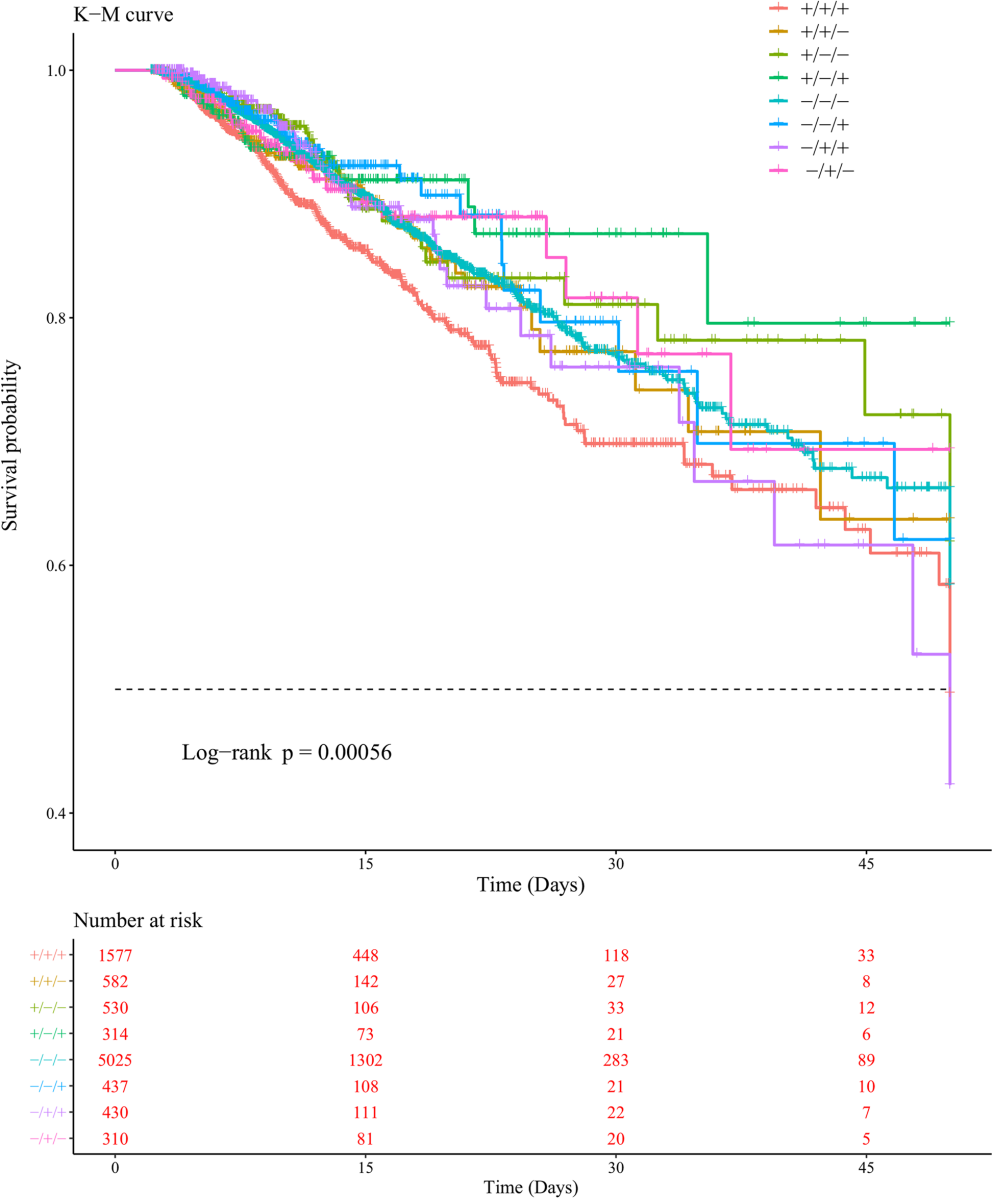
Relationship between fluid balance for three consecutive days and in-hospital mortality. The hazard ratios (HRs) and 95 % confidence intervals (error bars) were calculated from the COX regression, the adjustment factors are consistent with Fig. 2

**Fig. 5:**
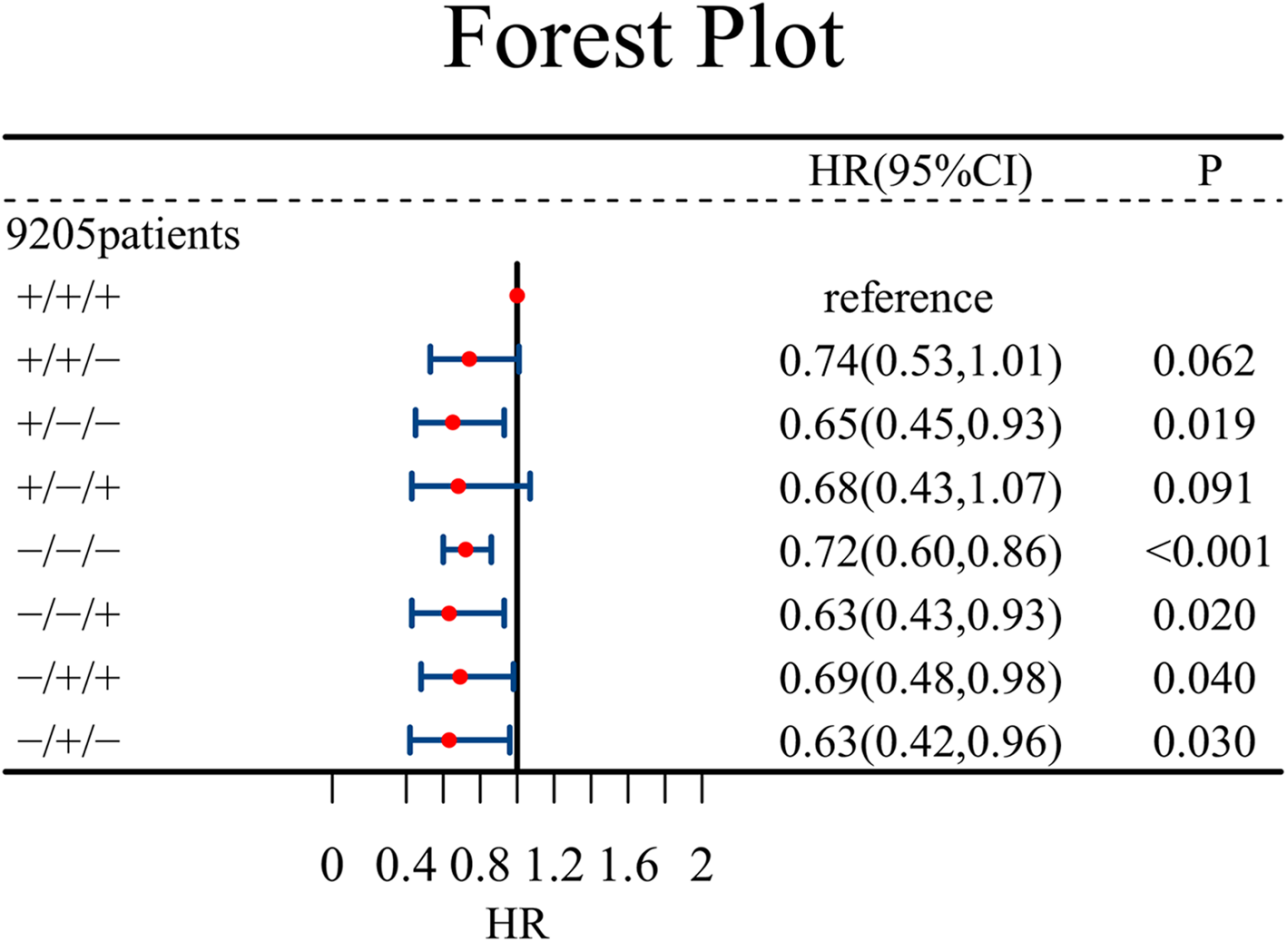
Kaplan–Meier curves revealed survival differences among patients in the eight groups

We selected a total of 9205 patients with fluid data for three consecutive days to further verify the effect of daily fluid balance on outcomes and divided them into the “+/+/+”, “+/+/−”, “+/ −/−”, “+/ −/+”, “−/ −/−”, “−/ −/+”, “−/+/+”, and “−/+/−” eight groups in accordance with their daily fluid balance. The Kaplan–Meier curve is shown in Fig. 4. After log-rank test, the P value was found to be less than 0.05, which indicated survival differences among patients in different groups. After adjustment for confounding factors, the Cox proportional hazard regression models showed that each group had different effects on outcomes. Compared with the “+/+/+” group, the “+/ −/−”, “−/ −/−”, “−/ −/+”, “−/+/+”, and “−/+/−” groups had a lower risk of in-hospital mortality, with HRs of 0.65 (0.45,0.93), 0.72 (0.60,0.86), 0.63 (0.43,0.93), 0.69 (0.48,0.98), and 0.63 (0.42,0.96), respectively, as shown in Fig. 5.

### Secondary outcomes

Linear regression showed that breathing machine use days differed between groups. For days 2–5, the “+” group had longer actual ventilator use days than the “−” group. For patients with fluid balance for three consecutive days, the “+/+/−”, “+/ −/−”, “−/ −/−”, “−/ −/+”, and “−/+/−” groups had fewer actual ventilator days than the “+/+/+” group (Table 2).

**Table 2:** The linear relationship between the duration of ventilator use and each group

DAY	GROUP	Estimate	P
DAY 1	-	reference	
	+	-0.072	0.174
DAY 2	-	reference	
	+	0.140	0.042
DAY 3	-	reference	
	+	0.220	0.009
DAY 4	-	reference	
	+	0.410	<0.001
DAY 5	-	reference	
	+	0.241	0.039
DAY1~3	+/+/+	reference	
	+/+/-	-0.37	0.045
	+/-/-	-0.79	<0.001
	+/-/+	-0.19	0.400
	-/-/-	-0.33	0.003
	-/-/+	-0.40	0.045
	-/+/+	-0.24	0.242
	-/+/-	-0.51	0.028

## Discussion

The pathological characteristics of sepsis are the reduction of effective circulating blood volume and the insufficient perfusion of tissues and organs in the body [13]. Liquid resuscitation can increase tissue perfusion by increasing cardiac output; improving the microcirculation disturbance caused by pathogenic microorganisms, their toxins, and inflammatory mediators in the host body; and then reducing mortality [14, 15]. Therefore, early fluid resuscitation and active and effective fluid volume management are very important for the rescue and treatment of patients with sepsis. However, there is increasing evidence that positive fluid balance during treatment in patients with sepsis is associated with increased mortality. For example, in a large cohort of patients with sepsis, a high cumulative fluid balance on day 3 after admission to the ICU is independently associated with an elevated risk of death [16]. Another retrospective study showed that a positive fluid balance within 24 h is associated with an increased risk of death [17]. The SOAP study, a large multicenter study on sepsis, demonstrated that positive fluid balance is one of the strongest prognostic factors for death in patients with sepsis [18].

Our study, which included 19,557 patients in a multicenter database, revealed that positive fluid balance on days 1, 2, and 3 after sepsis diagnosis was associated with poor prognosis, and the RCSs showed an overall trend of increasing the risk of in-hospital mortality with increasing fluid balance. We grouped patients with fluid balance records for the previous three consecutive days in accordance with daily fluid balance given that fluid administration is continuous and dynamic to further study the influence of fluid balance on patient mortality in the hospital. Compared with those in the “+/+/+” group, patients in the “+/ −/−”, “−/ −/−”, “−/ −/+”, “−/+/+”, and “−/+/−” groups were less likely to die in the hospital. The possible mechanism is that the vascular endothelial permeability of patients with sepsis is increased, and the overloaded fluid extravasates to cause tissue and organ edema, which is not conducive to the recovery of organ function and ultimately affects prognosis [19]. Hypervolume may exacerbate capillary leakage in patients with septic shock, leading to pulmonary edema [20]. Positive fluid balance is closely related to the occurrence of acute kidney injury in patients with sepsis [21]. Our secondary outcomes showed that the ventilator use time of the PB group from days 2 to 5 but not on day 1 was longer than that of the NB group. Compared with the “+/+/+” group, the “+/+/−”, “+/ −/−”, “−/ −/−”, “−/ −/+”, and “−/+/−” groups had fewer actual ventilator days for the first three days. This result suggested that positive fluid balance may affect lung function. Thus, although patients with sepsis need prompt fluid resuscitation, adequate perfusion, rather than aggressive, prolonged, and uncontrolled fluid infusion, should be provided on the basis of hemodynamic responsiveness. Even in patients who respond to fluid shock therapy and have a considerable increase in cardiac output, subsequent fluid infusion does not appear to improve microcirculation. Moreover, rapid fluid supplementation in patients with sepsis has only transient hemodynamic effects partly due to sepsis-induced vascular dysfunction and paralysis, which should be corrected by the use of vasoactive drugs rather than repeated rapid fluid supplementation [22].

Patients receive excess fluid, resulting in fluid overload. In the middle and late stages of sepsis, the pathogenesis and course of sepsis in patients are complex, and fluid management may be affected by numerous factors, such as the patients’ basic physical conditions and complications, fluid types, and the target endpoint of fluid resuscitation. At the same time, a clear demarcation between the stages of shock does not exist. Therefore, in clinical practice, grasping the two aspects of adequate fluid resuscitation in the early stage and restricted fluid management in the late stage of fluid therapy remains difficult [23]. Fluid therapy is an important measure for improving the perfusion of tissues and organs, maintaining the circulation state of the body, and correcting the metabolic disorders of the body and remains an indispensable part of the treatment of patients with sepsis. Although early and adequate fluid resuscitation is still recommended especially for patients with septic shock who may require additional fluid to maintain circulation stability, it is not the same as simple massive fluid replacement.

### Strengths and limitations of the study

The advantage of this study is that the eICU-CRD is a multicenter database, and its large sample size provides strong evidence for our study. In addition, we grouped the patients in accordance with their fluid balance on the first three consecutive days after diagnosis to further explore the mortality of patients in different combinations. However, this study has some limitations because it only investigated the relationship between positive fluid balance and mortality. Whether this relationship is a simple association or a causal relationship is not clear, and further confirmation with a large sample of prospective studies is needed.

## Conclusions

In patients with sepsis, positive fluid balance on days 1, 2, and 3 was associated with adverse outcomes. For patients with fluid balance for three consecutive days, the “+/−/−”, “−/ −/−”, “−/−/+”, “−/+/+”, and “−/+/−” groups were less likely to die in the hospital than the “+/+/+” group. In the treatment of sepsis, reasonable fluid therapy should be used, and continuous positive fluid balance is not recommended.

## Data Availability

The datasets generated and/or analysed during the current study are available in the eICU Collaborative Research Database, https://eicu-crd.mit.edu/.

## Abbreviations

NB/−: negative balance
PB/+: positive balance
RCSs: restricted cubic splines
ICU: intensive care unit
eICU-CRD: eICU Collaborative Research Database
SQL: Structured Query Language
SOFA: Sequential Organ Failure Assessment Score
APACHE: acute physiology and chronic health evaluation scoring system
CHF: congestive heart failure
COPD: chronic obstructive pulmonary disease
HR: hazard ratio
CI: confidence interval
